# Arbitrary 3D Organic Mixed Ionic‐Electronic Conductor Architectures via Self‐Fusion of PEDOT:PSS Microfibers

**DOI:** 10.1002/advs.202516951

**Published:** 2025-12-23

**Authors:** Youngseok Kim, Jongwon Lee, Jiwoong Kim, Jung Il Yoo, Junggeon Park, Jaeyoung Lee, Heung Cho Ko, HyungJu Ahn, Myung‐Han Yoon

**Affiliations:** ^1^ Department of Materials Science and Engineering Gwangju Institute of Science and Technology (GIST) Gwangju 61005 Republic of Korea; ^2^ Department of Materials·Nano·Manufacturing, Convergence and Open Sharing System Chung‐Ang University Seoul 06974 Republic of Korea; ^3^ Young Engineering Science Gyeryong Chungnam 32804 Republic of Korea; ^4^ Industrial Technology Convergence Center Pohang Accelerator Laboratory, POSTECH Pohang Gyeongbuk 37673 Republic of Korea

**Keywords:** 3‐D structures, microfiber, organic mixed ionic‐electronic conductors, PEDOT:PSS, self‐fusion

## Abstract

In this research, a first‐of‐its‐kind fabricating strategy is reported that assembles arbitrary 3D organic mixed ionic‐electronic conductor (OMIEC) architectures using poly(3,4‐ethylenedioxythiophene):polystyrene sulfonate (PEDOT:PSS) microfiber building blocks. This approach exploits a water‐assisted self‐fusion process, in which adhesion can be modulated as reversible (PSS‐rich) or irreversible (PEDOT‐rich) self‐fusion depending on the post‐treatment condition of building blocks. Phenomenological characterization and structural analyses reveal that hydration‐induced swelling of hydrophilic PSS chains and crystalline π–π‐stacked PEDOT domains govern interfacial bonding. Using PEDOT:PSS microfibers as modular units, structures ranging from 2D mesh electrodes to centimeter‐scale free‐standing 3D architectures are demonstrated. The resulting microfiber network structures are mechanically robust under bending and folding in aqueous environments and exhibit a high volumetric capacitance. Furthermore, hydration reduces the elastic modulus by ≈80%, enabling soft, conformal adhesion onto wet and irregular surfaces without additional adhesives. Finally, “cut‐and‐stick” PEDOT:PSS mesh electrodes are fabricated as a proof‐of‐concept and employed for recording in vivo cardiac activities from rodent hearts with minimal motion artifacts, outperforming conventional rigid platinum electrodes. This self‐fusion strategy establishes a simple and scalable route for the first‐time construction of arbitrary 3D OMIEC architectures, opening new opportunities for multifunctional OMIEC platforms in bioelectronics and energy‐storage applications.

## Graphical abstract

A general fabricating strategy for arbitrary 3D organic mixed ionic‐electronic conductor architectures is reported using PEDOT:PSS microfiber building blocks. A water‐assisted self‐fusion process is successfully developed in which adhesion can be modulated as reversible (PSS‐rich) or irreversible (PEDOT‐rich) self‐fusion depending on the post‐treatment condition of building blocks.

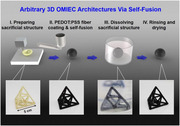

## 1 Introduction

Conducting polymers have long been recognized as promising materials for applications in energy conversion,^[^ ^1^, ^2^ ^]^ storage,^[^ ^3^, ^4^, ^5^ ^]^ and optoelectronics^[^ ^6^ ^]^ due to their intrinsic electronic conductivity, redox chemistry, mechanical flexibility, and processability. More recently, a specific class of conducting polymers—organic mixed ionic–electronic conductors (OMIECs)—has emerged as a cornerstone material platform for bioelectronics. Unlike conventional organic semiconductors that primarily conduct electronic charges, OMIECs can simultaneously transport electrons/holes and ions, enabling efficient ionic–electronic coupling at soft, aqueous interfaces. This mixed‐conduction capability makes them uniquely suitable for interfacing with bioelectrical systems, where ionic signals dominate.

Among OMIECs, poly(3,4ethylenedioxythiophene):polystyrene sulfonate (PEDOT:PSS) is the most widely studied and commercially available material. PEDOT:PSS combines high electronic conductivity^[^ ^7^ ^]^ and large volumetric capacitance^[^ ^8^, ^9^, ^10^ ^]^ with gel‐like softness,^[^ ^11^, ^12^, ^13^, ^14^, ^15^ ^]^ biocompatibility,^[^ ^16^ ^]^ and doping‐state tunability under electrochemical bias.^[^ ^17^, ^18^ ^]^ Owing to these properties, PEDOT:PSS has become a benchmark material for low‐impedance cellular interfaces^[^ ^19^, ^20^, ^21^ ^]^ and as the active channel layer in organic electrochemical transistors (OECTs) for bioelectronic applications such as electrophysiological recording^[^ ^22^, ^23^, ^24^, ^25^ ^]^ and chemical sensing.^[^ ^26^, ^27^, ^28^ ^]^ However, PEDOT:PSS is typically processed from aqueous dispersions, which restricts most device fabrication to solution‐based coating methods (e.g., spin‐coating, dip‐coating, bar‐coating, spray‐coating, and printing). As a result, PEDOT:PSS has predominantly been confined to 2D thin films deposited on rigid substrates. While suitable for certain electronic applications, such 2D architectures are inherently limiting for bioelectronic interfaces: in conjunction with underlying substrates, they restrict the accessible electrochemically active surface area, block mass transfer of biochemical species, and hinder seamless integration with underlying tissues.

To overcome these limitations, recent efforts have explored 3D PEDOT:PSS structures using various methods such as electrospinning,^[^ ^29^, ^30^, ^31^ ^]^ 3D printing,^[^ ^14^, ^32^, ^33^, ^34^, ^35^ ^]^ freeze‐drying,^[^ ^36^, ^37^, ^38^, ^39^, ^40^, ^41^ ^]^ and supercritical drying,^[^ ^42^ ^]^ which generate nano/microporous scaffolds that better mimic the in vivo environment. Additionally, PEDOT:PSS‐based 3D printing with viscous conducting gels has enabled stacked architectures with high aspect ratios.^[^ ^43^ ^]^ Nevertheless, these approaches remain constrained in scalability, shape complexity, and structural stability, largely due to the flowable nature of conducting inks and limited control over arbitrary geometries. In contrast, bulk‐scale engineering commonly relies on assembling modular building blocks with adhesives or fusion methods (e.g., concrete with cement, wires with solder, or metal frames with welding). Translating this principle to conducting polymers, the ability to mass‐produce uniform PEDOT:PSS building blocks and bond them reliably could provide a generic route to scalable, arbitrary 3D micro/microporous OMIEC architectures. This approach would avoid electrically insulating glues, which otherwise disrupt charge transport in electrically/ionically conductive networks and, possibly, cause biotoxicity‐related issues. Finally, it is highly desired that these 3D architectures should be decently adhesive as well as mechanically soft, leading to the conformal adhesion on biological tissues, while their electrical/electrochemical properties are well maintained.

In this research, we investigate the distinct reversible and irreversible self‐fusion behaviors of PEDOT:PSS microfibers and elucidate their molecular origin. Through a combination of phenomenological studies, X‐ray spectroscopy, and structural analyses, we demonstrate that the contrasting fusion behaviors arise from the interplay between hydration‐induced swelling of hydrophilic PSS chains and crystalline π–π‐stacked PEDOT domains. Based on this mechanistic understanding, we establish a first‐of‐its‐kind monolithic stacking strategy that exploits intrinsic self‐fusion to assemble short PEDOT:PSS microfibers (i.e., building blocks) into arbitrary 3D OMIEC architectures without the use of additional adhesives. Uniform building blocks were fabricated via a high‐throughput wet‐spinning and cutting process and subsequently assembled into free‐standing 2D mesh electrodes or complex 3D structures. The resulting architectures exhibit high electrical conductivity, large electrochemical capacitance, and mechanical robustness, while retaining foldability, cuttability, and scalability up to wafer scale. Moreover, their water‐induced modulus change enables rapid, conformal adhesion to irregular, hydrated biological surfaces. As a proof of concept, we demonstrate “cut‐and‐stick” PEDOT:PSS mesh electrodes for in vivo electrocardiography, which recorded stable cardiac signals from lively‐beating rodent hearts with minimal motion artifacts, outperforming conventional rigid platinum electrodes.^[^ ^44^, ^45^ ^]^

## 2 Results and Discussion

### 2.1 Reversible and Irreversible Self‐Fusion Behaviors of PEDOT:PSS Microfibers

**Figure 1:**
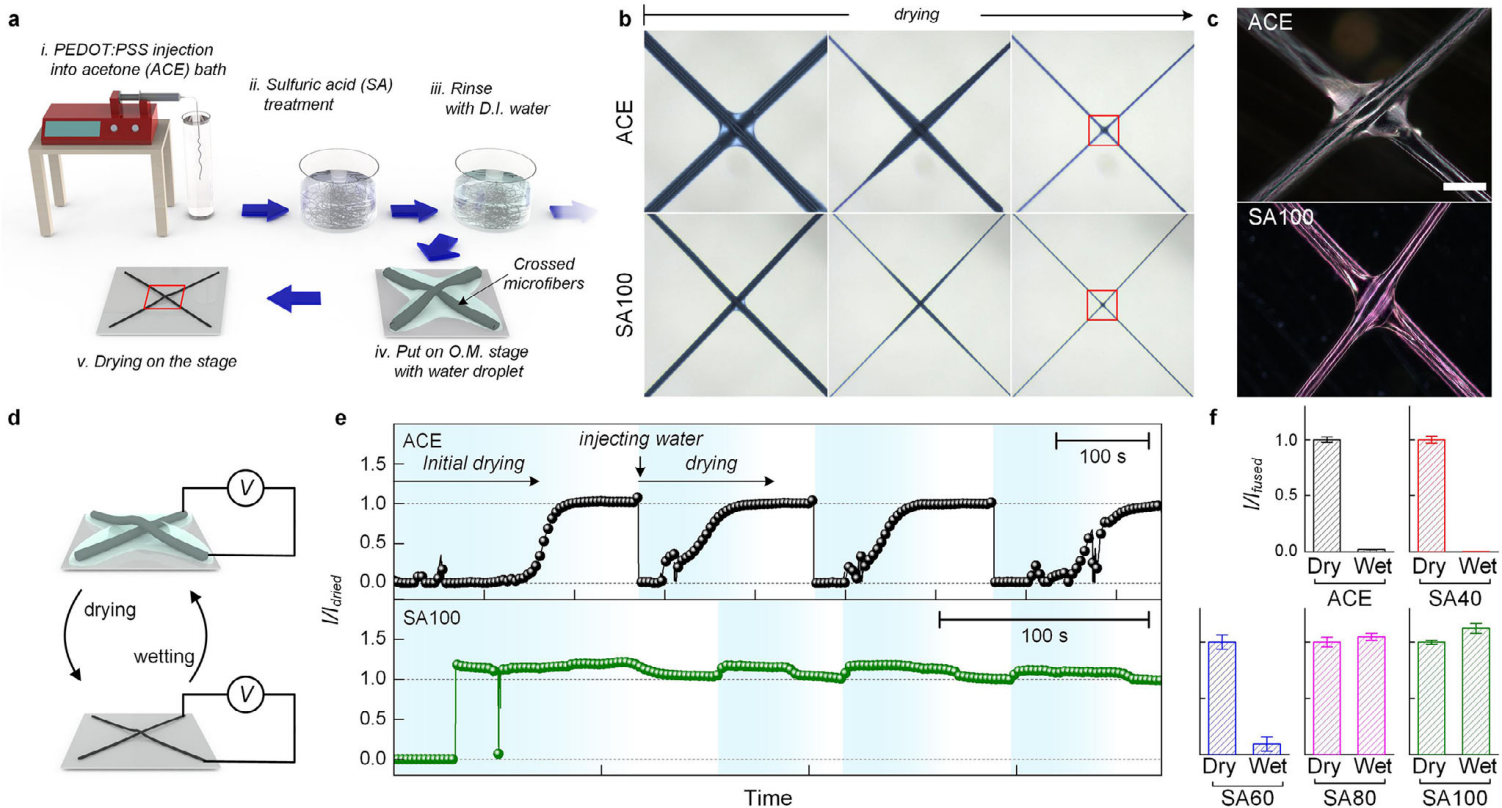
Phenomenological studies on the reversible and irreversible self‐fusion behaviors of PEDOT:PSS microfibers under different post‐treatment conditions. a) Schematic illustration of the fabrication of short PEDOT:PSS microfibers i–iii) and optical imaging of two crossed microfibers during drying. b) Time‐lapse optical microscopy images of two crossed microfibers during drying (scale bar: 200 µm). c) Polarized optical microscopy images of two crossed microfibers after complete drying (scale bar: 50 µm). In (b) and (c), the upper and lower rows correspond to ACE and SA100 PEDOT:PSS microfibers, respectively. d) Experimental scheme for in situ monitoring of electrical current through two microfiber junctions during drying and wetting. e) Plots of normalized current as a function of time during repeated drying/wetting cycles (ACE, upper; SA100, lower). f) Comparison of normalized currents between dry and wet two‐crossed‐microfibers prepared at different post‐treatment conditions (ACE, SA40, SA60, SA80, and SA100).

First, a distinct bonding phenomenon was observed between two PEDOT:PSS microfibers during the initial drying step. PEDOT:PSS microfibers were prepared by wet‐spinning into an acetone bath, followed by post‐treatment with aqueous sulfuric acid solutions of different concentrations and subsequent rinsing with water (**Figure** **1**a). For clarity, fibers were labeled according to the sulfuric acid concentration used for post‐treatment (i.e., SA100, SA80, SA60, and SA40 represent fibers treated with 100, 80, 60, and 40% sulfuric acid by volume in deionized water, respectively), while untreated fibers were denoted as ACE. This sulfuric acid treatment process induces phase separation between PEDOT and PSS domains and promotes crystallization of PEDOT domains, thereby improving electrical conductivity and structural integrity.^[^ ^46^ ^]^ When two fibers were crossed and dried together, they were seamlessly bonded at the intersection over time, suggesting spontaneous self‐fusion (Figure 1b,c; Figure S1, Supporting Information). Interestingly, upon re‐immersion in water, two contrasting outcomes were observed: fibers either separated due to excessive swelling or remained stably bonded with minimal swelling. Such a dramatic difference indicates that the nature of inter‐fiber bonding is strongly dependent on the residual PEDOT to PSS ratio.

To systematically evaluate this behavior, crossed microfiber pairs were placed on a microscope stage under hydrated conditions, with one end of each connected electrically. The electrical current flow across the junction was monitored at 10 mV bias during repeated drying/wetting cycles induced by the addition of water droplets (Figure 1d). After the initial drying, all fiber pairs formed both physical and electrical contacts, accompanied by volume shrinkage (Figure 1e; Figure S2, Supporting Information). Upon re‐hydration, ACE fibers and those treated with 40–60% sulfuric acid exhibited excessive expansion, leading to the loss of electrical contact. In contrast, fibers treated with ≥80% sulfuric acid maintained stable junctions, showing only minor current fluctuations. This behavior is attributed to reduced PSS content, enhanced PEDOT crystallinity, and doping stability after post‐treatment with highly concentrated (≥80%) sulfuric acid solutions, which suppresses excessive swelling of PSS chains while maintaining conductive pathways (Figure 1f).^[^ ^46^, ^47^ ^]^ Importantly, the resistance of adhered/elongated fibers formed by this self‐fusion process was nearly identical to that of a single fiber with the equal length, without significant additional contact resistance at the junction (Figure S3a, Supporting Information). Furthermore, multi‐stacked fibers exhibited predictable resistance scaling under parallel and series connections, confirming effective electrical integration (Figure S3b, Supporting Information).

For comparison, the same experiment was performed on two dried fibers, which were brought into contact solely by external pressure (Figure S4a, Supporting Information). Increasing the applied vertical force up to ≈50 N produced only partial electrical contact with the current plateauing at 40–50% of that of a single fiber (Figure S4b, Supporting Information). This poor junction formation is attributed to the rough surface morphology of dried fibers and their relatively high modulus (≈1 GPa), which restricts the effective contact area despite partial collapse under load (Figure S4c, Supporting Information).

### 2.2 What Causes PEDOT:PSS Microfibers to Exhibit Distinct Reversible and Irreversible Self‐Fusion Behaviors

**Figure 2:**
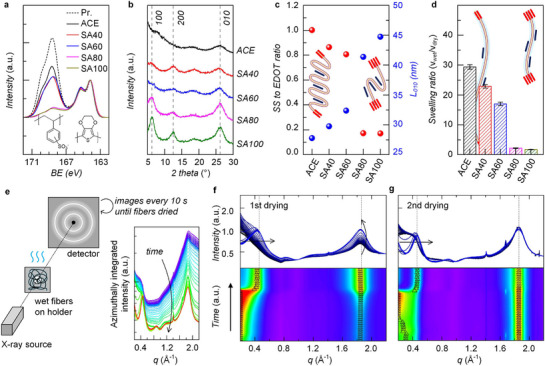
In‐depth investigation of the mechanisms underlying reversible and irreversible self‐fusion behaviors. a) XPS and b) XRD spectra of PEDOT:PSS microfibers depending on post‐treatment condition (ACE, SA40, SA60, SA80 and SA100). c) SS to EDOT ratios (left; red circles) and crystalline domain size of PEDOT π–π stacking (*L*_010_, right; blue circle) extracted from XPS and XRD, respectively. Inset: schematic illustration of PSS chains crosslinked by π–π stacked PEDOT crystalline domains in PSS‐rich (left) and PEDOT‐rich (right) cases after drying. d) Swelling ratios of PEDOT:PSS microfibers depending on post‐treatment condition. Inset: schematic illustration of PSS chains crosslinked by π–π stacked PEDOT crystalline domains in PSS‐rich (left) and PEDOT‐rich (right) cases under wet conditions. e) Experimental scheme for *in‐operando* Tr‐WAXD measurements. f,g) Spectral changes during the first and second drying processes of wet PEDOT:PSS microfibers depending on post‐treatment condition.

To elucidate the origin of the reversible versus irreversible self‐fusion of PEDOT:PSS microfibers, their chemical composition and microstructure were analyzed by X‐ray photoelectron spectroscopy (XPS) and X‐ray diffraction (XRD). As shown in the sulfur 2*p* peak spectra, the ratio of sulfonate (SS, from PSS) to thiophene (EDOT, from PEDOT) groups decreased markedly with increasing sulfuric acid concentration for post‐treatment, from 1.0 in ACE to 0.17 in SA100 (**Figure** **2**a). This reduction can be attributed to the protonation of the sulfonic acid group in PSS upon sulfuric acid treatment, which weakens electrostatic interactions between PSS and PEDOT chains and facilitates selective removal of unbound PSS from the as‐prepared PEDOT:PSS.^[^ ^7^ ^]^ In parallel, XRD measurements revealed that the lamellar stacking peak at 6.25° and its second‐order peak at 12.5° intensified, while the π–π stacking peak at 26° also increased from ACE to SA100 (Figure 2b). Consequently, the average PEDOT grain size increased from 27 (ACE) to 45 nm (SA100), consistent with fewer PSS chains, which hinder PEDOT crystallization (Figure 2c). To explain the above results, we argue that PEDOT:PSS can be viewed as a moderately‐swelling hydrogel system: PSS chains act as hydrophilic polymer segments and PEDOT π–π stacked domains serve as cross‐linking points. The swelling ratio of hydrogels is governed by the average chain length between crosslinking points (inset, Figure 2c). In parallel, swelling measurements showed the same trend: the swelling ratio decreased dramatically with reduced SS/EDOT ratio, from 29‐fold (ACE) to onefold (SA100) (Figure 2d). This result suggests that as the PSS fraction decreases and PEDOT crystallinity increases with stronger sulfuric acid treatment, the effective length of hydrophilic PSS chains shortens, leading to reduced swelling (inset, Figure 2d).

Next, in‐operando transmission wide‐angle X‐ray diffraction (Tr‐WAXD) was performed to monitor structural changes during the first and second drying steps. To isolate the PEDOT:PSS signal from water scattering, each spectrum obtained from hydrated microfibers was deconvoluted using a reference spectrum from a pure water droplet (Figure 2e). Figure 2f,g represent the spectra during the first and second drying processes, respectively, with black indicating the wet state and blue the fully dried state. In the never‐dried condition (black line in Figure 2f), the lamellar stacking peak appeared at *q* < 0.2 Å^−1^, indicating large interchain spacing (>3.1 nm), while the π–π stacking peak at 1.85 Å^−1^ showed very low intensity. Upon drying, the lamellar peak shifted to *q* ≈ 0.4 Å^−1^ (corresponding to shorter spacing of 1.6 nm) with increased intensity, accompanied by a stronger π–π stacking peak. When the fibers were re‐swollen, the lamellar peak shifted back below *q* = 0.2 Å^−1^, reflecting chain expansion, whereas the π–π stacking peak remained unchanged. Importantly, after the second drying cycle (blue line, Figure 2g), all peaks returned to the positions and intensities of the first dried state (blue line, Figure 2f). Considering the hydrophilic nature of PSS and the hydrophobic nature of PEDOT, these results indicate that PSS‐based lamellar crystalline domains are easily distorted and reversibly expanding upon water re‐penetration, whereas PEDOT‐based π–π crystalline domains remain stable and their expansion is irreversible.

**Scheme 1:**
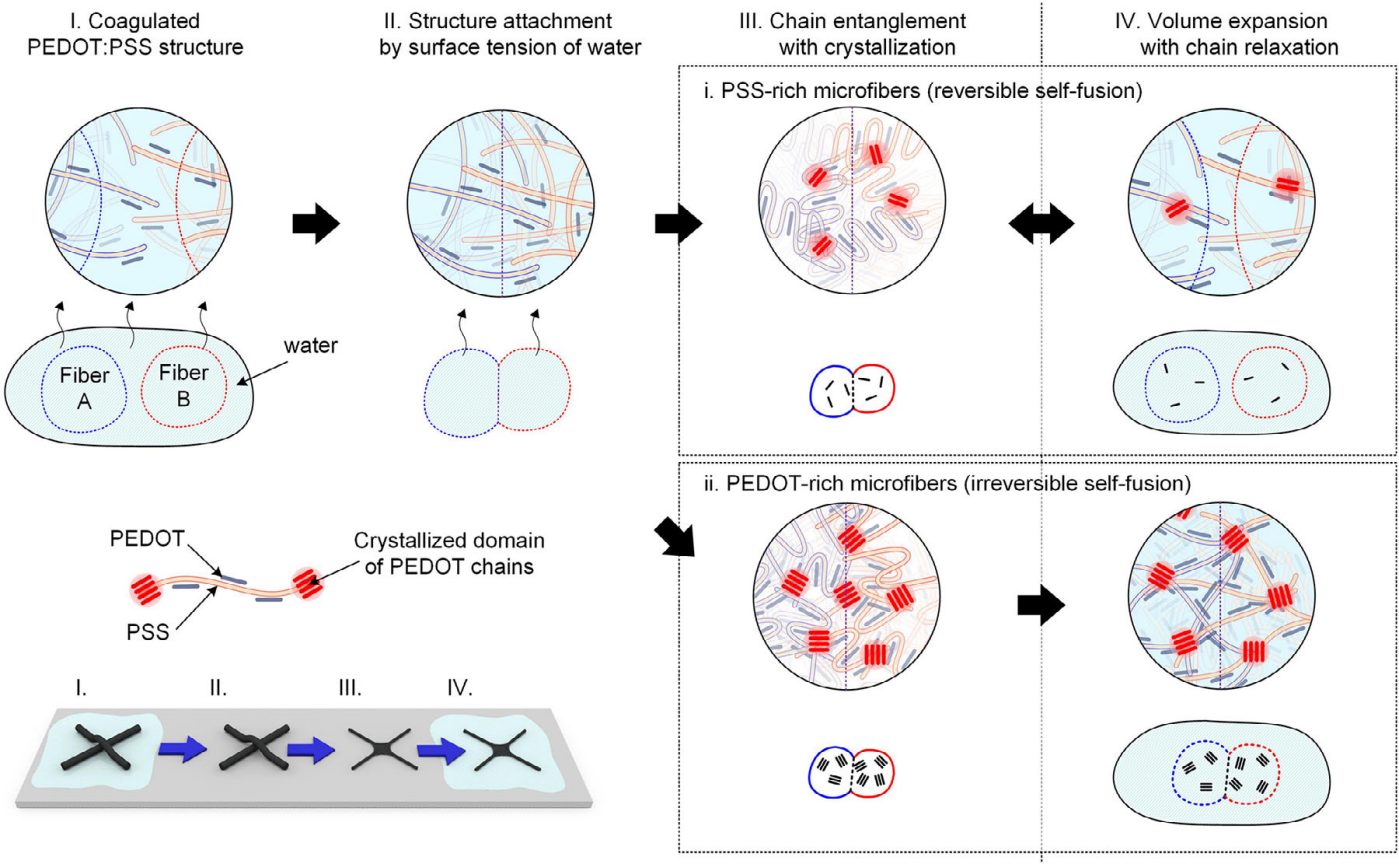
Schematic illustration of i) reversible and ii) irreversible self‐fusion processes of PSS‐rich and PEDOT‐rich microstructures, respectively.

Based on the phenomenological observations and microstructural analyses described above, the self‐fusion of PEDOT:PSS microfibers can be categorized into two distinct cases: i) reversible self‐fusion for PSS‐rich fibers (e.g., ACE, SA40, and SA60) and ii) irreversible self‐fusion for PEDOT‐rich fibers (e.g., SA80 and SA100). Step‐by‐step molecular schematic models for both cases are summarized in **Scheme** **1**. In the hydrated state (Step I), all PEDOT:PSS microfibers exhibit gel‐like solid structures, since their solubility decreases after coagulation in acetone and further PSS removal during sulfuric acid post‐treatment. During drying (Step II), surface water evaporates first, leading to the partial collapse of adjacent microfibers driven by surface tension and the formation of capillary bridges. Upon complete dehydration (Step III), polymer chains from neighboring fibers interpenetrate, entangle, and crystallize, generating lamellar domains and π–π stacked PEDOT regions across the interface. For PSS‐rich fibers (Case i, Steps III–IV), the long hydrophilic PSS chains and smaller crystalline cross‐linking domains enable excessive swelling upon re‐exposure to water. This swelling disrupts chain entanglement and distorts PEDOT crystallites, weakening the inter‐fiber junctions. However, during subsequent drying, the untangled PSS chains can re‐entangle, allowing the self‐fusion process to remain reversible. In contrast, PEDOT‐rich fibers (Case ii, Steps III–IV) contain shorter PSS segments and larger π–π cross‐linking domains. These structural features suppress swelling and stabilize inter‐fiber junctions through irreversible PEDOT–PEDOT stacking that persists even after re‐hydration. As a result, PEDOT‐rich microfibers exhibit strong, water‐stable adhesion following the first drying step, accompanied by minimal volume expansion in aqueous environments during re‐hydration. Note that the proposed mechanism of self‐fusion between PEDOT:PSS microfibers is inspired by that of the self‐healing in PEDOT:PSS films reported in the previous literature.^[^ ^48^ ^]^ This self‐fusion behavior primarily originates from the crystallization of hydrophobic domains (i.e., π–π stacking of PEDOT chains) during the drying process, while hydrophilic domains (i.e., PSS chains) solvated with water molecules induce reflowing and mixing. Accordingly, if i) a material contains both hydrophobic moieties that can act as cross‐linking sites and hydrophilic moieties that can induce swelling/reflowing/mixing in the presence of solvent, and ii) the material undergoes crystallization upon solvent removal, this self‐fusion strategy could potentially be extended to other OMIEC systems as well.

### 2.3 Formation of 2D and 3D Meshed Structures From a Long Single Microfiber via Self‐Fusion

**Figure 3:**
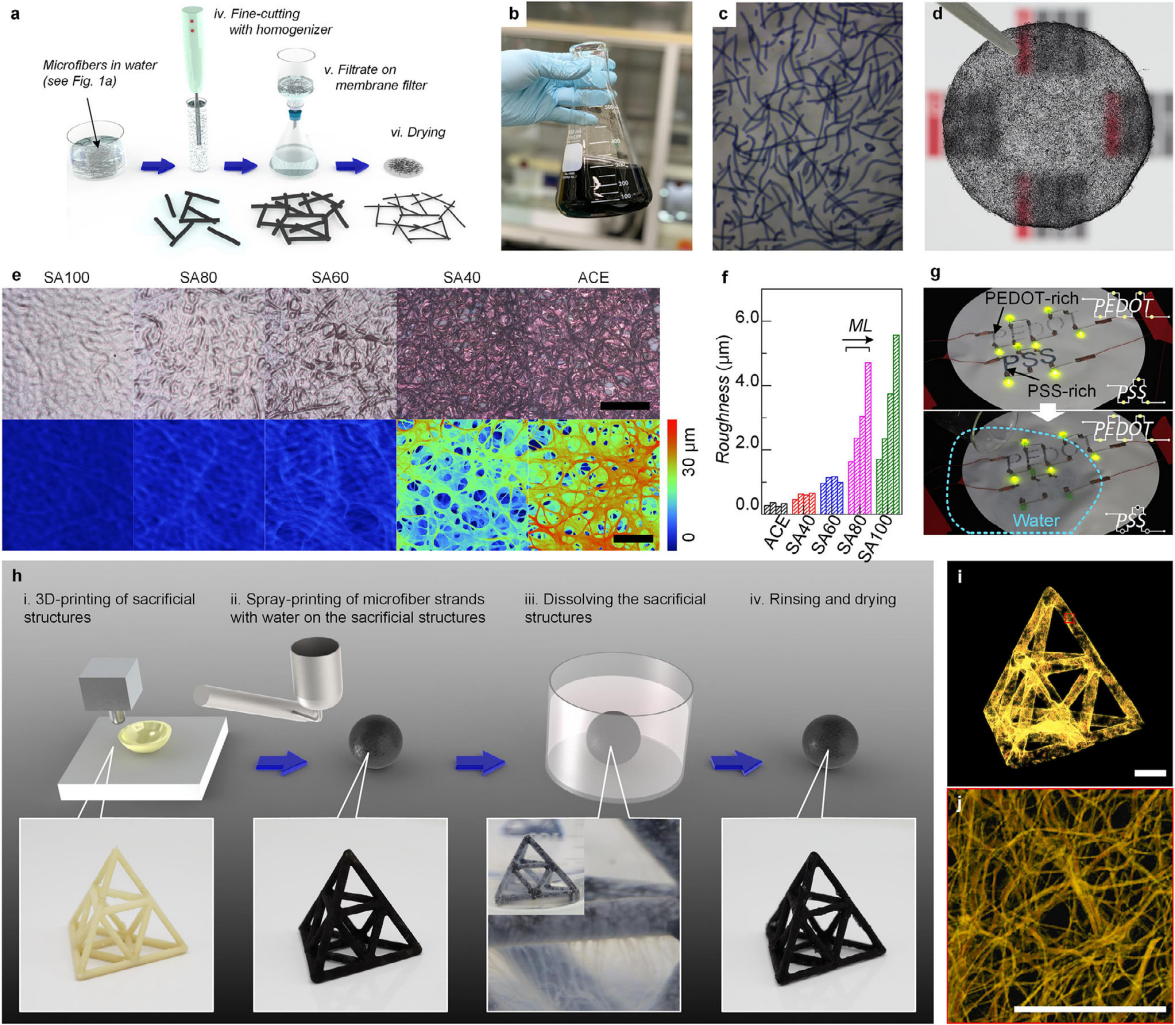
Fabrication of multi‐dimensional PEDOT:PSS microfiber network structures. a) Schematic illustration of the fabrication of PEDOT:PSS microfiber mesh electrodes by cutting and assembling the wet‐spun fibers shown in Figure 1a, followed by irreversible self‐fusion. b) Photograph and c) microscopic images of short PEDOT:PSS microfibers (SA100) after homogenization. d) As‐prepared PEDOT:PSS mesh electrodes. Scale bar denotes 10 mm. e) Optical microscopy images (upper) and optical profilometry images (lower) of PEDOT:PSS mesh electrodes prepared with ACE, SA40, SA60, SA80, and SA100. Scale bars denote 200 µm. f) Plots of average roughness of PEDOT:PSS mesh (ACE, SA40, SA60, SA80, and SA100) as a function of mass loading. g) Patterned PEDOT:PSS mesh electrodes prepared by stencil masking. (Upper) The as‐prepared “PEDOT” and “PSS” letters were defined with PEDOT‐rich and PSS‐rich PEDOT:PSS microfibers, respectively. LEDs were connected in series through the letters, while a 5 mA current was applied. (Lower) The “PEDOT”/”PSS” with LED and power supply were immersed in water. Scale bars denote 50 mm. h) Schematic illustration of step‐by‐step fabrication of 3D PEDOT:PSS microfiber architectures. A sacrificial structure was prepared (step i) and spray‐coated with short PEDOT:PSS microfiber dispersion, followed by self‐fusion via drying water (step ii). Subsequently, the inner sacrificial scaffold was dissolved with acetone (step iii), yielding 3D PEDOT:PSS microfiber architectures without any adhesive. i) Reconstructed tomographic image of the overall 3D tetrahedral architectures with j) magnified image of the region marked with red square in (i). Scale bars denote 10 mm.

The self‐fusion behavior described above was employed to fabricate multi‐dimensional PEDOT:PSS architectures with high versatility. To achieve monolithic stacked structures, three requirements were identified: i) a large number of small building blocks must be generated from a single long PEDOT:PSS microfiber, ii) the blocks should remain in a never‐dried state prior to self‐fusion, and iii) the first drying step must be intentionally induced at desired positions. The fabrication process for producing building blocks and multi‐stacked 2D structures is outlined in **Figure** **3**a. Following wet‐spinning, post‐treatment, and rinsing (Figure 1a), long PEDOT:PSS microfibers were cut into short fibers using a mechanical homogenizer under shear force. These short fibers with a few millimeters in length and a few hundred micrometers in diameter served as building blocks (Figure 3b). Due to the intrinsic hydrophilicity of PEDOT:PSS, the resultant short fibers remain dispersed stably in water (Figure 3c). For 2D stacked films, they were collected onto cellulose acetate membrane filters by vacuum filtration, followed by complete dehydration in a vacuum oven. After peeling from the filter, the resulting free‐standing films displayed interconnected mesh‐like structures formed by spontaneous self‐fusion between PEDOT:PSS microfibers (Figure 3d).

In parallel, optical microscopy and profilometry revealed that ACE, SA40, and SA60 short fibers collapsed extensively during drying, forming dense films, whereas SA80 and SA100 samples maintained junction‐only fusion, yielding microporous pseudo‐3D networks (Figure 3e). Indeed, surface roughness measurements confirmed the same trend (Figure 3f). 2D mesh films prepared with SA80 and SA100 fibers exhibited much greater roughness, reflecting the robustness of PEDOT‐rich strands during drying. Furthermore, the roughness of those prepared with SA80 and SA100 samples increased steeply with fiber loading, while those prepared with ACE, SA40, and SA60 samples showed that roughness remained insensitive to mass loading, indicative of the progressive stacking into in‐plane meshed architectures. Interestingly, the water‐dispersible nature of the as‐prepared short PEDOT:PSS microfibers also enabled the spray‐based patterning. Using stencil masks, the patterned mesh electrodes spelling “PEDOT” (with SA100 fibers) and “PSS” (with ACE fibers) were fabricated (Figure 3g). LEDs connected in series through each pattern lit up under a 5 mA bias. Remarkably, upon water exposure, the PEDOT‐rich electrodes (i.e., “PEDOT”) maintained electrical continuity and kept the LEDs illuminated, while the PSS‐rich electrodes (i.e., “PSS”) disintegrate, extinguishing the LEDs. This simple demonstration highlights the material‐dependent water stability of PEDOT‐rich versus PSS‐rich microfibers, as well as the versatility of self‐fusion for patterned device fabrication.

For 3D network architecture construction, short PEDOT:PSS microfibers (SA100) were sprayed onto acrylonitrile butadiene styrene (ABS)‐based sacrificial 3D scaffolds prepared by conventional 3D printing, followed by drying (Figure 3h, steps i–ii). Subsequent immersion in acetone completely dissolved the sacrificial core, yielding free‐standing centimeter‐scale 3D PEDOT:PSS microfiber network architectures (Figure 3h, step iii). The porous nature of the self‐fused microfiber mesh facilitated solvent penetration, ensuring complete scaffold removal (Figure 3h, step iii, inset). X‐ray tomography confirmed the absence of internal ABS (Figure 3i), and the external PEDOT:PSS layer was revealed to form a randomly oriented, multi‐stacked microfiber network (Figure 3j). The fabricated 3D architectures exhibited superior structural and electrical characteristics in comparison with 3D architectures prepared by electrospinning, 3D printing, or freeze‐drying methods (Table S1, Supporting Information). It is noteworthy that this is the first demonstration of arbitrary centimeter‐scale 3D architectures constructed entirely from PEDOT:PSS—or any organic mixed ionic–electronic conductor—achieved through self‐fusion alone, without the need for electrically insulating glues or adhesives.

### 2.4 Electrical, Electrochemical, and Mechanical Properties of PEDOT:PSS Microfiber Mesh Structures

**Figure 4:**
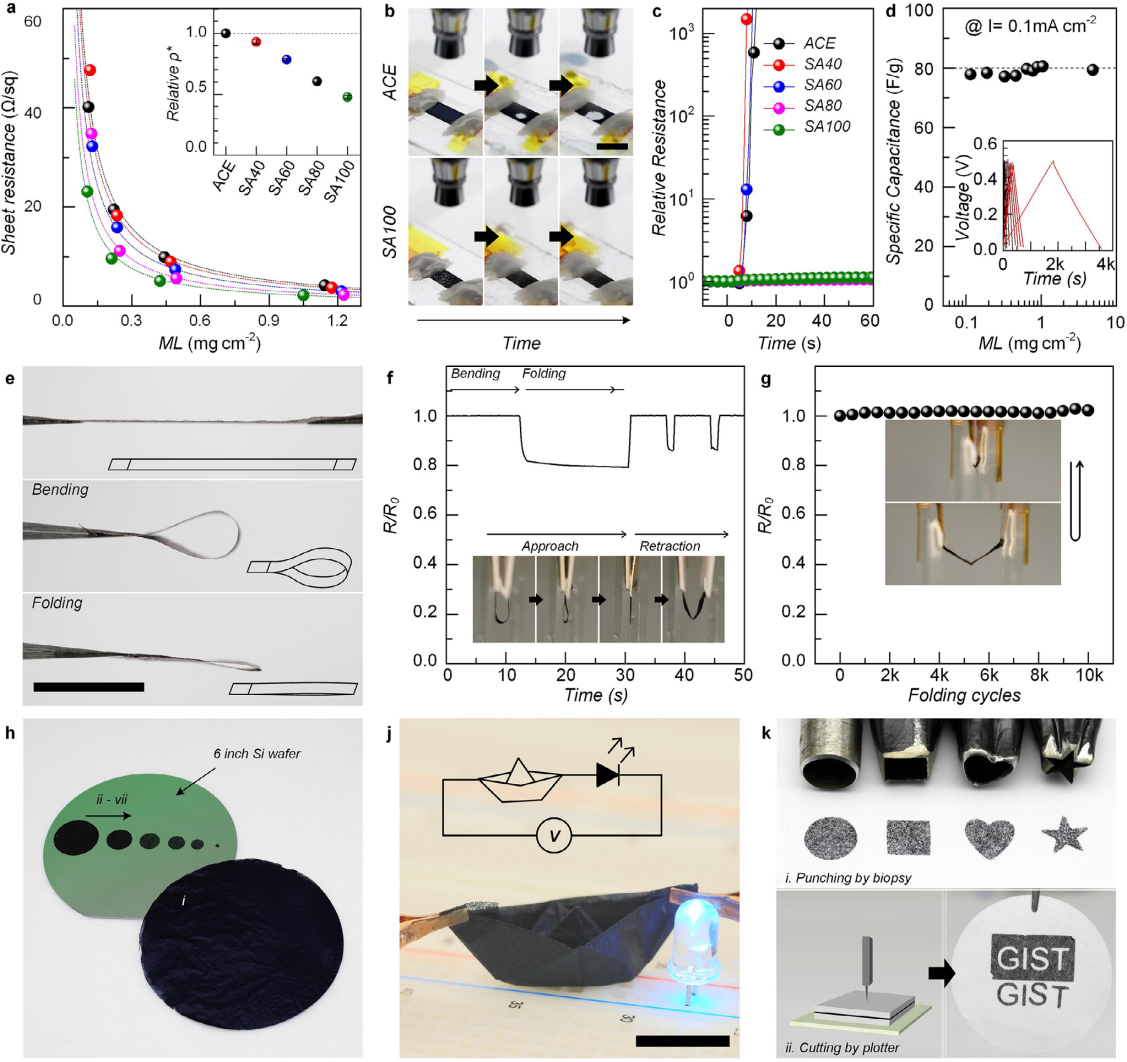
Electrical, electrochemical, and mechanical properties of PEDOT:PSS mesh electrodes. a) Plots of sheet resistance of self‐fused ACE, SA40, SA60, SA80 and SA100 mesh as a function of mass loading. b) Snapshots of ACE and SA100 mesh under water‐jet spraying. Scale bar denotes 10 mm. c) Plots of relative resistance of self‐fused mesh as a function of exposure time to water‐jet spraying. d) Gravimetric capacitance of SA100 mesh electrodes in comparison with PEDOT:PSS thin films. e) Photographic images of PEDOT:PSS mesh electrodes: (upper) free‐standing, (middle) bent, and (lower) folded: Scale bar denotes 10 mm. f) Relative resistance changes during bending and folding. Mesh electrodes were bent with radii decreasing from 5.0 to ≈0.0 mm. g) Relative resistance variations over 10 000 folding cycles. h) Photographs of PEDOT:PSS mesh electrodes with various sizes. The diameter of circular electrodes ranged from 152.4 mm (6 inch) to 3 mm. j) Photographs of a origami ship made by folding 2D PEDOT:PSS mesh. Inset: equivalent circuit diagram. Scale bar denotes 50 mm. k) Patterned PEDOT:PSS mesh electrodes fabricated by i) punching with various biopsy tools and ii) cutting with a plotter machine.

**Figure** **4**a shows the sheet resistance of PEDOT:PSS microfiber mesh electrodes as a function of post‐treatment and mass loading (ML). Note that the electrical properties of self‐fused PEDOT:PSS microfiber mesh electrodes were evaluated in terms of sheet resistance rather than conductivity, as the porous laminated fiber structure makes it difficult to define an accurate thickness. Electrodes prepared with SA100 short microfibers displayed significantly lower relative specific resistivity (ρ* = *ρ* × ML; Figure 4a inset) than those prepared with ACE, which is in good agreement with theoretical 1/ML fitting curves (dashed lines). This behavior is consistent with the previously known effect of sulfuric acid treatment in enhancing PEDOT conductivity in thin films and fibers.^[^ ^46^, ^49^ ^]^ Unlike conventional PEDOT:PSS films, however, the SA100 mesh electrodes maintained conductivity even under severe hydraulic stress. When exposed to a water‐jet stream, ACE mesh electrodes rapidly failed due to puncturing from the stream center, thus, a sharp increase in resistance (Figure 4b,c). In contrast, SA100 mesh electrodes remained intact, exhibiting negligible resistance changes after >1 min of direct water spraying. Note that intermediate SA40/SA60 and SA80 samples behaved similarly to the ACE and SA100 samples, respectively, underscoring the composition‐dependent stability under severe hydraulic stress. Such irreversible self‐fusion driven by crystalline PEDOT endows the mesh electrodes with excellent aqueous stability. Even after three weeks of immersion in water, the SA100 mesh electrodes exhibited no significant degradation and, in fact, showed decreased resistance (Figure S5, Supporting Information). This behavior (i.e., increased conductance) can be attributed to the denser packing of PEDOT chains, as the polymer network repeatedly loosened and reorganized during the testing process. Moreover, it is remarkable that the fabricated 3D PEDOT:PSS microfiber architectures, when stored in water, have preserved their robust network structure even after five years, thereby confirming their outstanding long‐term structural stability (Figure S6, Supporting Information).

Electrochemical properties were also evaluated by galvanostatic charging–discharging (Figure 4d). The gravimetric capacitance (*C*_A_ = *C*/*A*) increased linearly with ML, yielding a slope of 76.5 F g^−1^. Considering that the previously known density of PEDOT:PSS is 1.06 g cm^−^^3^, the volumetric capacitance was calculated as 81.1 F cm^−^^3^, which is lower than that of a PEDOT:PSS single microfiber prepared by the same method (122 F cm^−^^3^). We expect that differences in the characterization method, use of an imprecise density parameter, and, in particular, moisture absorbed during weighing may lead to an underestimation of the volumetric capacitance. Even with these differences, the nearly constant volumetric capacitance as a function of mass loading clearly indicates that the bulk mesh structure, featuring seamless bonding among PEDOT:PSS microfibers, exhibits outstanding electochemical performance without compromising doping efficiency, in contrast to foams or aerogels, where structural heterogeneity and reduced ion mobility in the bulk often reduce the resultant volumetric capacitance.

From the mechanical perspective, the composition‐wise homogeneous substrate‐free mesh electrodes place the neutral mechanical plane within the active material itself, which grants exceptional deformability. In fact, the resultant PEDOT:PSS microfiber mesh electrodes could be easily manipulated, bent, and folded without damage by hand or tweezers (Figure 4e). The corresponding resistance remained unchanged even with a bending radius approaching 0 mm (Figure 4f), and the same metric increased by only 3.1% even after 10 000 folding cycles (Figure 4g). The slight resistance drop observed during the first folding could possibly be attributed to the improved interfacial contact between folded planes. The 3.1% increase in resistance observed after 10 000 folding cycles could be attributed to deformation of the contact regions caused by the repeated folding process. Such durability far exceeds that of PEDOT:PSS thin films, which typically crack under comparable strain. The observed mechanical resilience may be attributed to two features, as supported by our previous research: i) PEDOT:PSS chains within pleats re‐align under deformation, partially compensating for resistance increases,^[^ ^49^ ^]^ and ii) individual microfibers tolerate elongations of 40–50% since they are produced without prestrain during wet‐spinning.^[^ ^50^ ^]^

Scalability and versatility are also important toward broad‐impact electrode systems for versatile bioelectronic interfaces and energy storage devices. Large‐area mesh electrodes up to 6 inches in diameter were readily fabricated by simple vacuum filtration (Figure 4h). In addition, their foldability enabled origami structures as demonstrated by the origami ship functioning as a conductive electrode to power LEDs (Figure 4i,j). In addition, the mesh electrodes could be easily patterned by scissors, biopsy punches, or cutting plotters with sub‐millimeter resolutions. Letters “G,” “I,” “S,” and “T” were patterned with a critical dimension of 0.9 mm, producing sharply defined meshes with high contrast (Figure 4k). Note that such ease of post‐fabrication shaping and miniaturization is rarely possible with conventional PEDOT:PSS films or foams, which easily fracture, delaminate, and/or lose electrical continuity.

### 2.5 Water‐Induced Switchable Mechanical Modulus

**Figure 5:**
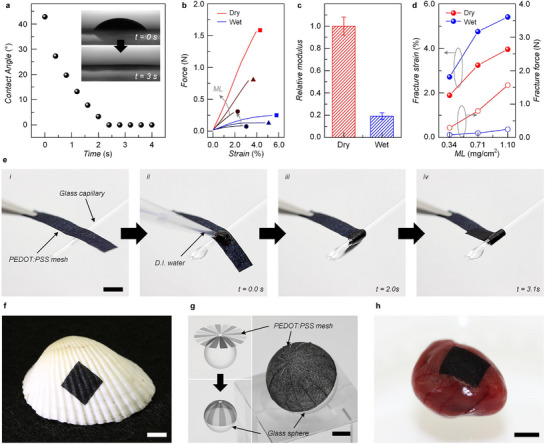
Water‐induced switchable mechanical properties of PEDOT:PSS mesh electrodes. a) Plot of water contact angle of PEDOT:PSS mesh electrodes as a function of time. Inset: snapshot images taken immediately after water droplet contact (upper) and after 3 s (lower). b) Force‐strain curves of PEDOT:PSS mesh electrodes with different mass loadings (0.34 mg cm^−2^, circle; 0.71 mg cm^−2^, triangle; 1.1 mg cm^−2^, square) before (dry; reddish) and after water exposure (wet; bluish). c) Relative modulus of mesh electrodes before (dry; reddish) and after water exposure (wet; bluish). d) Plots of fracture strain (left, solid symbols) and fracture force (right, open symbols) as a function of mass loading. e) Time‐lapse photographs of a rectangular mesh electrode in contact with glass rod after water exposure. Scale bar denotes 5.0 mm. Photographs of PEDOT:PSS mesh electrodes attached onto f) a seashell, g) glass spheres (see the main text for details), and h) a rodent heart. All scale bars denote 5 mm.

Bioelectronic interfaces inevitably operate in aqueous environments—such as sweat and ambient moisture in vitro, or blood and interstitial fluids in vivo—where stable device performance requires materials with adaptable mechanical properties. PEDOT:PSS microfiber mesh electrodes prepared by the irreversible self‐fusion (e.g., SA100) are particularly suitable for such conditions. To assess their mechanical adaptability, the wettability and modulus‐switching behavior of SA100 mesh electrodes were characterized in water. First, the water contact angle measurements confirmed immediate wetting: owing to the hydrophilic PSS chains and abundant microcapillaries in the porous mesh, water droplets were fully absorbed within 3 s, with the contact angle decreasing from 42 to 0° (**Figure** **5**a). Force–strain measurements of dried (reddish) and wet (bluish) mesh electrodes at different fiber mass loadings (ML = 0.34 (circle), 0.71 (triangle), and 1.1 mg cm^−^^2^ (square)) are shown in Figure 5b. Note that since the exact cross‐sectional area of the porous mesh is difficult to define, direct comparison between dried and wet samples with identical ML was employed. In the case of dried mesh electrodes, the slope of the force‐strain curve increased with higher ML, mainly due to the increased thickness; however, wet mesh electrodes consistently showed lower modulus than dried counterparts. The relative mechanical moduli, which were obtained by dividing the slope of the force‐strain curve by the corresponding ML revealed an ≈80% decrease in modulus upon hydration, attributed to water penetration into PSS chains, which weakens PEDOT–PSS lamellar stacking (Figure 5c). Considering that the typical modulus of an individual PEDOT:PSS microfiber is ≈1 GPa,^[^ ^49^ ^]^ the hydrated mesh electrodes reach several hundred MPa, a range comparable to biological tissues. Fracture strain and force analysis further demonstrated that, upon hydration, the PEDOT:PSS microfiber meshes become softer and more ductile (Figure 5d).

This water‐enabled modulus switching allows the free‐standing mesh electrodes to attach conformally to moisturized or biological surfaces with irregular topography (i.e., water‐assisted adhesion). As illustrated in Figure 5e, mesh electrodes initially rigid in the dry state can be easily handled and positioned with tweezers i); upon hydration, water rapidly permeates the porous network and polymer chains by capillary effect and moderate swelling, softening the mesh, thus enabling conformal adhesion onto and wrapping around a glass rod within 3 s (ii → iv). Similarly, wet mesh electrodes seamlessly cover rough shell surfaces (Figure 5f). Moreover, when combined with cutting‐plotter patterning, mesh electrodes could be transferred onto spherical substrates (radius = 20 mm) via a water‐printing process (Figure 5g), a task difficult to achieve with conventional thin‐film bioelectronic devices. Furthermore, mesh electrodes conformally cover the irregular wet surface of a biological tissue/organ, as demonstrated in Figure 5h.

### 2.6 “Cut‐and‐Stick” PEDOT:PSS Mesh Electrode for In Vivo Electrocardiography

**Figure 6:**
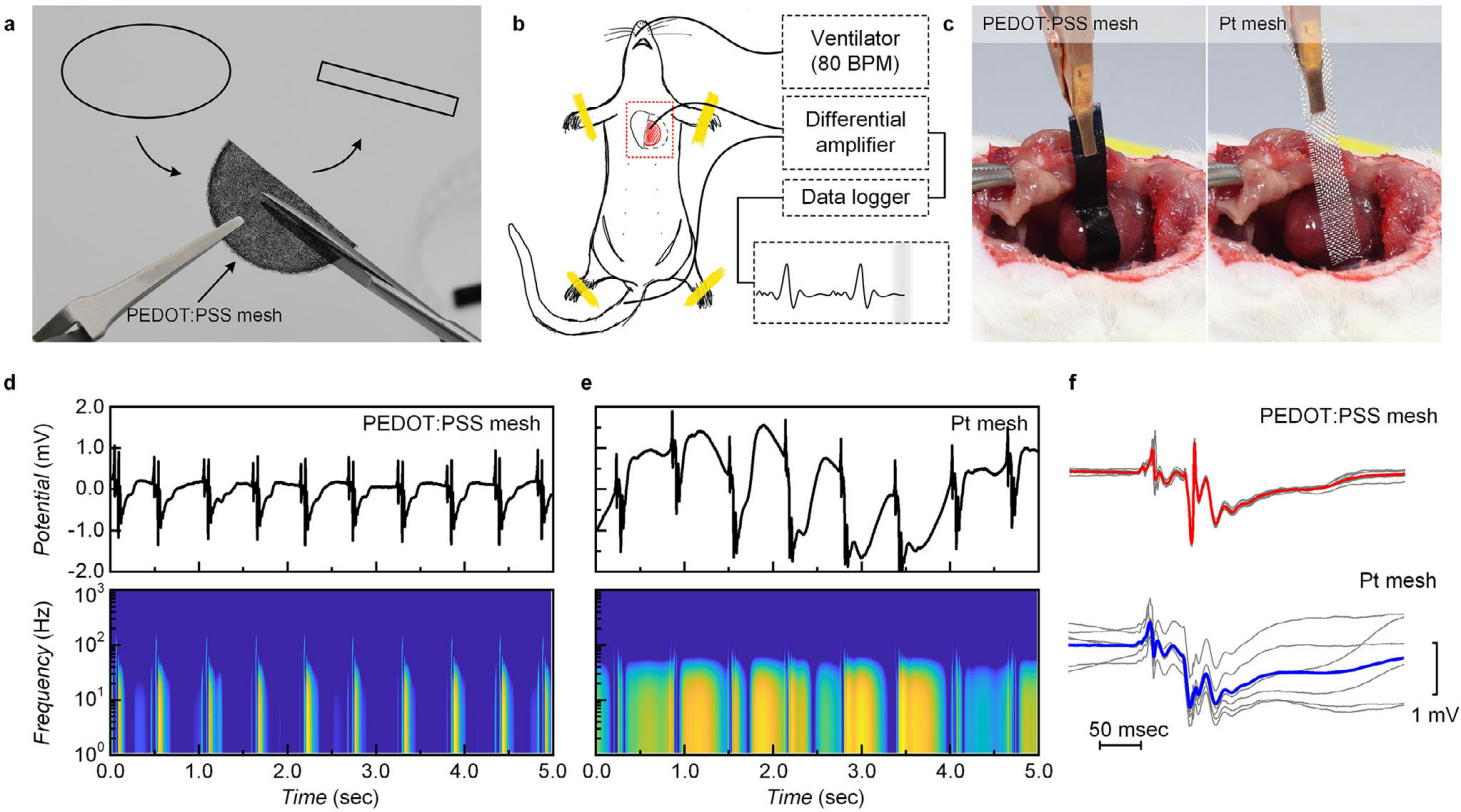
“Cut‐and‐stick” PEDOT:PSS mesh electrodes for in vivo electrocardiography (ECG). a) Schematic illustration of the preparation of PEDOT:PSS bioelectrodes. A rectangular PEDOT:PSS mesh electrode (35 mm × 5 mm) were shaped with scissors. b) Schematic illustration of ECG recording from a rodent heart. During the experiments, the animal was ventilated at 80 BPM and electrophysiological signals were acquired using a differential amplifier and data logger. c) Photographs of PEDOT:PSS mesh electrodes (left) and conventional Pt mesh electrodes (right) placed on the lively beating heart during measurements. d,e) Electrophysiological signal traces (upper) and corresponding power spectra (lower) acquired from PEDOT:PSS mesh electrodes and conventional Pt mesh electrodes. f) Acquired and sorted extracellular field potentials recorded from PEDOT:PSS mesh electrodes (upper) and conventional Pt mesh electrodes (lower). Scale bars denote 50 ms (*x*‐axis) and 1 mV (*y*‐axis).

To leverage the water‐enabled modulus switching for bioelectronics, PEDOT:PSS mesh electrodes were employed as sensing interfaces to record electrocardiography (ECG) signals from live rodent hearts. Prior to surgery, circular PEDOT:PSS meshes (prepared via vacuum filtration; see also Figure 3a,d) were cut into a rectangular shape (5 mm × 25 mm; **Figure** **6**a) and connected to an amplifier and data logger using copper clips (Figure 6b). Subsequently, the resultant “cut‐and‐stick” mesh electrode was then transferred onto the surgically opened chest and conformally attached to the right ventricle via simple water‐assisted adhesion. For comparison, identical measurements were performed using conventional platinum (Pt) mesh electrodes, which exhibit excellent electrical and electrochemical stability. PEDOT:PSS mesh electrodes were seamlessly attached onto the wet tissue surface, adherent through water‐assisted surface tension while vibrating synchronously with cardiac motion (Figure 6c, left). Note that a larger and more stable contact area is uniquely enabled by the soft, porous architecture of PEDOT:PSS microfiber mesh. In contrast, the rigid Pt mesh with the same shape failed to form intimate contact with the curved heart surface in vibrating motion, adhering only partially through mechanical bending (Figure 6c, right). During recording, the electrodes slipped and vibrated with each heartbeat, producing unstable contacts.

In fact, the effect of intimate electrode–tissue adhesion is reflected in the recorded signal quality (Figure 6d,e). Even without post‐processing such as band‐pass or notch filtering, softened PEDOT:PSS mesh electrodes directly recorded clean field potential traces with distinct spectral features below 100 Hz. In contrast, rigid Pt mesh electrodes generated severe motion artifacts, dominated by cardiac contractions and ventilator‐induced breathing (≈1.33 Hz), resulting in blurred spectra. This difference in data quality is also clearly evident in the signals processed with a Butterworth filter (Figure S7, Supporting Information). However, as is common in signal processing, the output signal is highly dependent on the selected cutoff frequency and filtering order, resulting in operator dependence. It is noteworthy that because ECG signals arise from muscle‐cell activity, they are particularly susceptible to motion artifacts caused by electrode displacement and interface friction, and such contamination is intrinsic to rigid metal systems and cannot be eliminated without extensive filtering.^[^ ^51^ ^]^

PEDOT:PSS mesh electrodes overcome these limitations by combining electronic/ionic conductivity with mechanical compliance unattainable with other OMIECs and inorganic systems. Their modulus in the hundreds of MPa range allows them to deform with biological tissues while maintaining adhesion and conductivity. As a result, the “cut‐and‐stick” PEDOT:PSS mesh electrodes stably captured field potentials with minimal artifacts (Figure 6f, upper), in sharp contrast to the noisy signals from Pt mesh electrodes (Figure 6f, lower). This demonstration highlights, for the first time, that arbitrary, self‐fused PEDOT:PSS mesh electrodes enable robust, high‐fidelity bioelectronic interfaces on soft, lively moving organs—performance unattainable with conventional thin‐film OMIECs or rigid metal electrodes.

## 3 Conclusions

This research establishes the principle and utility of self‐fusion in PEDOT:PSS microfibers as a generic strategy for constructing arbitrary 2D and 3D organic mixed ionic–electronic conductor (OMIEC) architectures. The comprehensive and systematic analyses revealed that inter‐fiber bonding originates from a combination of water‐induced surface tension and subsequent crystallization of PEDOT chains, with the balance between PEDOT‐rich and PSS‐rich domains dictating reversible versus irreversible self‐fusion. PSS‐rich microfibers enabled repeatedly reversible bonding and fabrication of vertically compact sheet electrodes, while PEDOT‐rich microfibers formed irreversible junctions with robust crystalline domains, yielding mechanically and electrically stable architectures with large porosity. The resulting PEDOT:PSS meshes combined durability (3.1% resistance change after 10 000 bending cycles), high volumetric capacitance (81.1 F cm^−^^3^), and unique water‐enabled modulus reduction (≈80%), which brought the mechanical compliance of the material into the range of biological tissues. Furthermore, these properties enabled the fabrication of free‐standing, large‐area PEDOT:PSS mesh electrodes and centimeter‐scale 3D architectures without additional adhesives, which are not straightforward to realize with conventional OMIECs or rigid metal/inorganic electrodes. As a proof of concept, conformal “cut‐and‐stick” mesh electrodes reliably recorded in vivo cardiac signals from lively beating rodent hearts with minimal motion artifacts, highlighting their exceptional capability as soft, tissue‐conforming bioelectronic interfaces. Remarkably, the self‐fusion concept offers a versatile route toward organ‐ and tissue‐customized implantable bioelectronics. The excellent biocompatibility, aqueous stability, and high electrical conductivity of PEDOT:PSS, combined with its porous mesh‐like architecture, make it a highly promising material for stable and functional operation in chronic bioelectronic applications, fulfilling the essential requirements of long‐term implantable interfaces. By tailoring geometry, porosity, and bonding characteristics, future devices could integrate seamlessly with curved and dynamic organs, not only for stable electrophysiological recording but also for localized electrical stimulation and therapeutic modulation. Ultimately, such advances may pave the way for next‐generation bioelectronic platforms that merge structural adaptability, electrical performance, and long‐term stability to interface directly with complex biological systems.

## 4 Experimental Section

### Materials

Aqueous PEDOT:PSS solution (PH1000) was purchased from Heraeus. Acetone (EP grade) and sulfuric acid (EP grade) were obtained from Duksan Chemical. Cellulose acetate membrane filters were purchased from Hyundai Micro.

### Preparation of PEDOT:PSS Microfibers

Aqueous PEDOT:PSS solution was filtered through a syringe filter (Sartorius, 17593) and injected into an acetone coagulation bath using syringe pumps (New Era Pump Systems, NE‐300) equipped with 30G needles at a flow rate of 500 µL h^−1^. The coagulated microfibers were rinsed with deionized water and treated with sulfuric acid solutions (40%, 60%, 80%, and 100%) in an orbital shaker for 12 h. The fibers were then rinsed thoroughly with deionized water until the solution pH reached ≈7. For ACE fibers, the sulfuric acid treatment step was omitted. For phenomenological studies, thicker fibers were prepared using the same setup but with a spinning rate of 2 mL h^−1^.

### Preparation of PEDOT:PSS Mesh Electrodes

PEDOT:PSS microfiber bundles were rinsed with deionized water and finely cut using a homogenizer (Daihan Scientific, HG‐15D) at 1000 rpm for 60 s. The resulting short microfibers dispersed in water were vacuum‐filtered onto cellulose acetate membranes and dried in a vacuum oven (60 °C, ≈10 mTorr) for 10 min. Fully self‐fused PEDOT:PSS mesh electrodes were detached in deionized water, collected, and dried with a nitrogen gun.

### Material Characterization

XPS analysis was conducted on a K‐Alpha XPS instrument (Thermo Fisher Scientific). Samples were prepared by pulverizing frozen mesh sheets in liquid nitrogen with a mortar and pestle. All spectra were normalized to the S 2p peak of PEDOT. XRD was performed using an Empyrean diffractometer (PANalytical) with a Cu radiation source. As‐prepared mesh sheets were used directly, and spectra were normalized to the minimum and maximum intensity. Tr‐WAXD measurements were carried out at the 9A U‐SAXS beamline of PLS‐II (Pohang, Republic of Korea). The sample‐to‐detector distance and X‐ray wavelength were set to 221 mm and 1.12 Å, respectively. The diffraction angle was calibrated using a precalibrated sucrose standard (monoclinic, P2_1_, a = 10.8631 Å, b = 8.7044 Å, c = 7.7624 Å, and β = 102.938°).

### Phenomenological Study Using Microfibers

For in situ structural and electrical monitoring, two PEDOT:PSS fibers were crossed on a microscopy stage and electrically connected at both ends to copper electrodes. As swollen fibers dried on the electrodes, they collapsed and adhered, forming a junction. Current through the junction was measured under a 10 mV bias during drying. For re‐swelling, ≈200 µL water droplets were added to the junction until volume expansion was saturated.

### Geometrical and Topological Characterization

Optical microscopy images were obtained using a stereoscopic microscope (Sunny Optical Technology, SZMN) with a CCD camera or an optical microscope (Olympus BX51). 3D surface profiles were acquired using an optical profiler (Nanofocus µSurf). Images were processed with ImageJ, Gwyddion, or Photoshop. Line profiles and roughness were measured using a surface profiler (Bruker, DektakXT).

### Electrical, Electrochemical, Mechanical, and Optical Characterization

Sheet resistance was measured by a four‐point probe system with a source measure unit (Keithley 2400) under a 1 mA bias. Electrochemical capacitance was extracted from galvanostatic charge–discharge tests using an electrochemical workstation (Metrohm Autolab PGSTAT 302N) with an Ag/AgCl reference electrode, Pt mesh counter electrode, and PEDOT:PSS mesh working electrode immersed in 100 mm NaCl electrolyte. Samples were prepared by biopsy punching (diameter = 8 mm). Capacitance was normalized to area, and sample masses were measured using a microbalance (Radwag MYA 2.4Y) for gravimetric normalization. Mechanical properties were measured with a dynamic mechanical analyzer (TA Instruments DMA 2980). Specimens (width = 5 mm, length = 10–15 mm) were fixed at both ends with epoxy (Alteco F‐301). Cyclic bending and folding were tested using a custom Arduino‐controlled linear stage, while resistance was monitored with a digital multimeter (Keithley 2100).

### Water‐Jet Stability Test

Mesh electrodes (width = 5 mm, exposed length = ≈8 mm) were attached to glass slides (Paul Marienfeld) with double‐sided adhesive. Ends were connected to Cu wires using Ag paste (CANS Elcoat P‐100) and sealed with dielectric epoxy (Alteco F‐301). A vertical water jet was applied from 20 mm at 200 kPa pneumatic pressure, while a current under 10 mV bias was recorded using a Keithley 2400 controlled with custom MATLAB code.

### Fabrication of 3D Architectures

Sacrificial scaffolds were printed using a 3D printer (Sindoricoh 3DWOX 2X) with ABS filaments. PEDOT:PSS microfibers were spray‐coated onto the scaffold using a spray coater (Beetle Bug GP70) and dried with a 70 °C heat gun (Bosch GHG16‐50). After coating, the ABS core was dissolved in acetone with sonication, followed by rinsing in isopropanol and deionized water, and drying with nitrogen.

### Cutting and Origami Demonstrations

Mesh electrodes were mounted on glass carrier substrates (Corning Eagle XG) with poly(vinyl alcohol)‐based water‐soluble tape and patterned using a cutting plotter (Silhouette Cameo3). Processed samples were immersed in ≈60 °C water for 1 h to dissolve the tape, and the patterned meshes were collected on filter paper. Square sheets (5 × 5 mm^2^) were used for origami demonstrations.

### ECG Recording

All animal experiments were approved by the Committee on Animal Research and Ethics at Gwangju Institute of Science and Technology, Republic of Korea (Approval number: GIST 2021–026). Sprague Dawley rats were anesthetized via intraperitoneal injection of ketamine (70 mg kg^−1^, Yuhan) and xylazine (15 mg kg^−1^, Bayer) in saline. Ventilation was performed with a polyethylene catheter (4G) using a rodent ventilator (Harvard Apparatus, USA) set at 80 bpm. After thoracotomy, the ribs were fixed with a retractor, and electrodes were placed on the heart surface. For in vivo ECG, a needle electrode was inserted into the tail as a reference, while a Pt electrode or PEDOT:PSS mesh electrode was placed on the right ventricle. Signals were amplified with a differential amplifier (A‐M Systems Model 1800) and recorded using a data acquisition system (Molecular Devices Axon Digidata 1500). No digital filtering (e.g., band‐pass, DC removal) was applied. Power spectra were obtained using MATLAB.

## Conflict of Interest

The authors declare no conflict of interest.

## Supporting information



Supporting Information

## Data Availability

The data that support the findings of this study are available from the corresponding author upon reasonable request.
