# Supplementary material for: Arbitrary 3D Organic Mixed Ionic‐Electronic Conductor Architectures via Self‐Fusion of PEDOT:PSS Microfibers
Source: Adv Sci (Weinh). 2025 Dec 23;13(10):e16951. doi: 10.1002/advs.202516951 (PMC12915079; doi:10.1002/advs.202516951)
Supplement: Supplementary file 1 — Supporting Information [file ADVS-13-e16951-s001.pdf]

Supporting Information

Arbitrary 3D Organic Mixed Ionic–Electronic Conductor Architectures via Self-Fusion of PEDOT:PSS Microfibers

Youngseok Kim[1,2,3], Jongwon Lee[1], Jiwoong Kim[1], Jung il Yoo[1], Junggeon Park[1], Jaeyoung Lee[1], Heung Cho Ko[1], HyungJu Ahn[4*], and Myung-Han Yoon[1]**

[1]School of Materials Science and Engineering, Gwangju Institute of Science and Technology, Gwangju 61005, Republic of Korea

[2]Department of Materials·Nano·Manufacturing, Convergence and Open Sharing System, Chung-Ang University, Seoul 06974, Republic of Korea

[3]Young Engineering Science, Gyeryong, Chungnam 32804, Republic of Korea

[4]Industrial Technology Convergence Center, Pohang Accelerator Laboratory, POSTECH, Pohang, Gyeongbuk 37673, Republic of Korea

*Corresponding author: Dr. Youngseok Kim, Dr. HyungJu Ahn, and Prof. Myung-Han Yoon

E-mail: youngs.kim@young-eng-sci.com, hyungju@postech.ac.kr, mhyoon@gist.ac.kr

Keywords: organic mixed ionic-electronic conductors, PEDOT:PSS, microfiber, 3-D structures, self-fusion

Table S1. Comparison among representative methods for 3D OMIEC structures

**Method**	**Resolution**	**Conductivity**	**Stability**	**References**
Electrospinning	100 nm – 10 μm (fiber size)	0.1 – 100 S/cm	Good (5 - 100 % strain)	[30],[31],[32]
3D printing	10 μm – 1 mm (layer thickness)	1 – 1000 S/cm	Good (Shape retention)	[14],[33],[34], [35],[36]
Freeze-drying	10 – 100 μm (pore size)	0.01 – 20 S/cm	Good (Shape retention)	[37],[38],[39], [40],[41],[42]

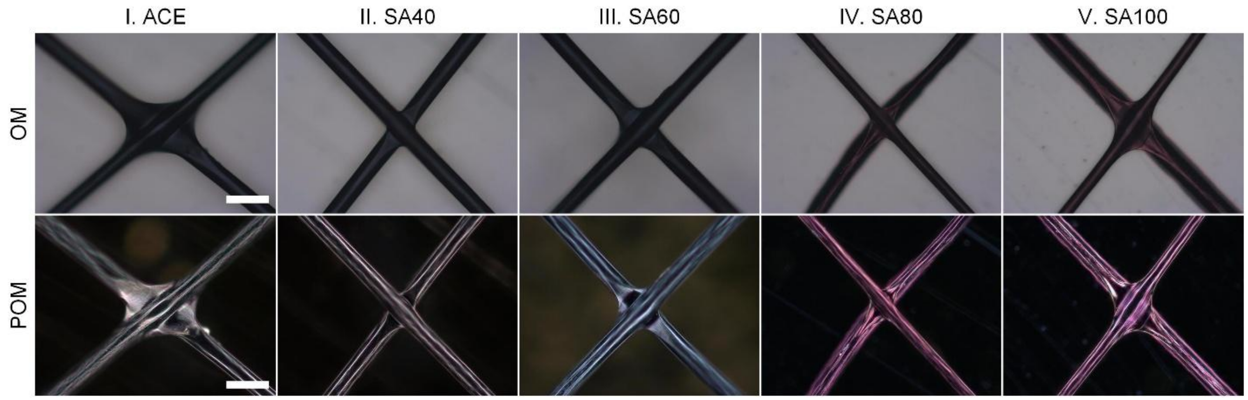

Figure S1. 1 Optical microscopy image of self-fused crossed PEDOT:PSS microfibers (ACE, SA40, SA60, SA80, SA100) without (upper) and with polarizer (lower). Scale bar denotes 50 μm.

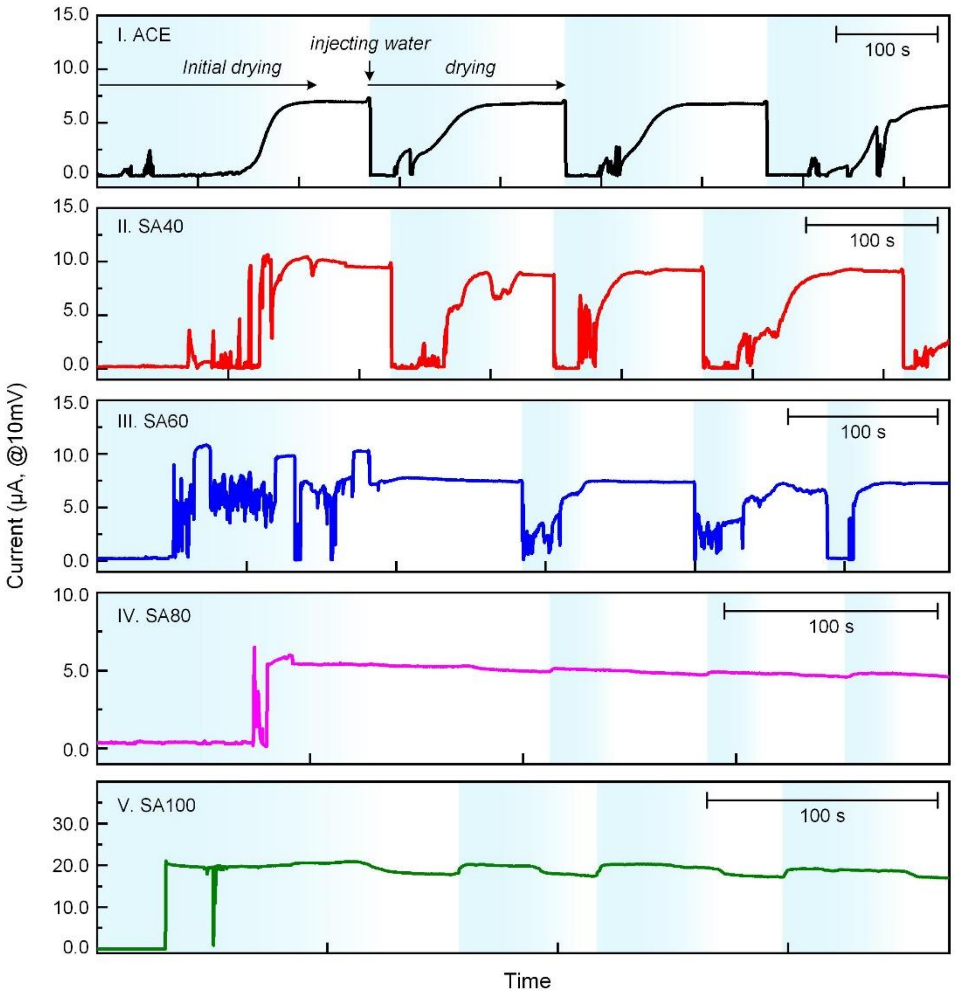

Figure S2. Plots of current through self-fused microfiber junction over time during repeated drying/wetting cycles. Each scale bar denotes 100 s.

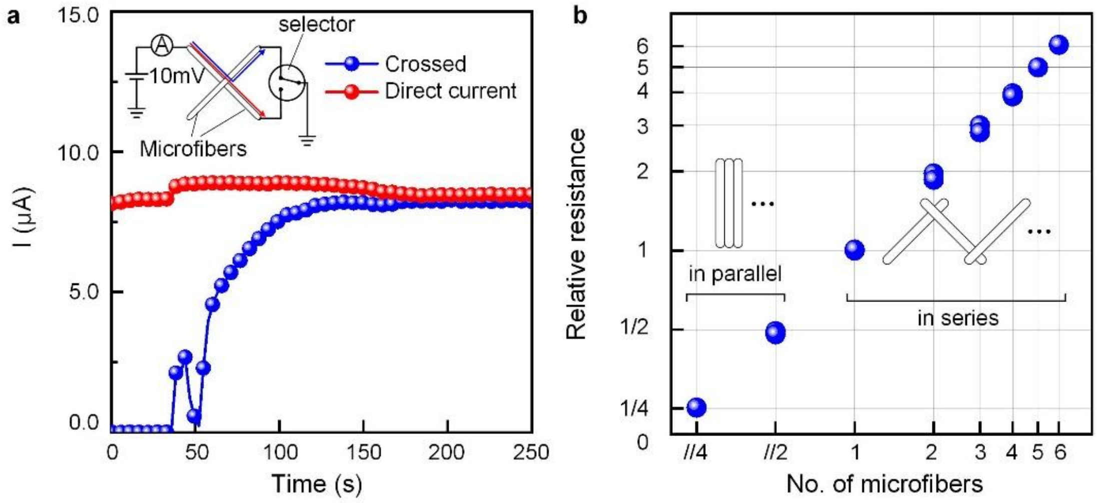

Figure S3. (a) Plots of current through a single microfiber (red, direct current) and two self-fused microfibers (blue, crossed current) over time during drying. As shown in the measurement scheme (inset), the total lengths of a single microfiber and two self-fused microfibers were identically set ~15 mm, and each current value (at 0.1 V bias) was continuously acquired with switching circuit-embedded source-measure unit (1 sec interval). (b) Relative resistance in self-fused microfibers which were connected in parallel and in series. The measured resistance was normalized with that of a single microfiber without junction.

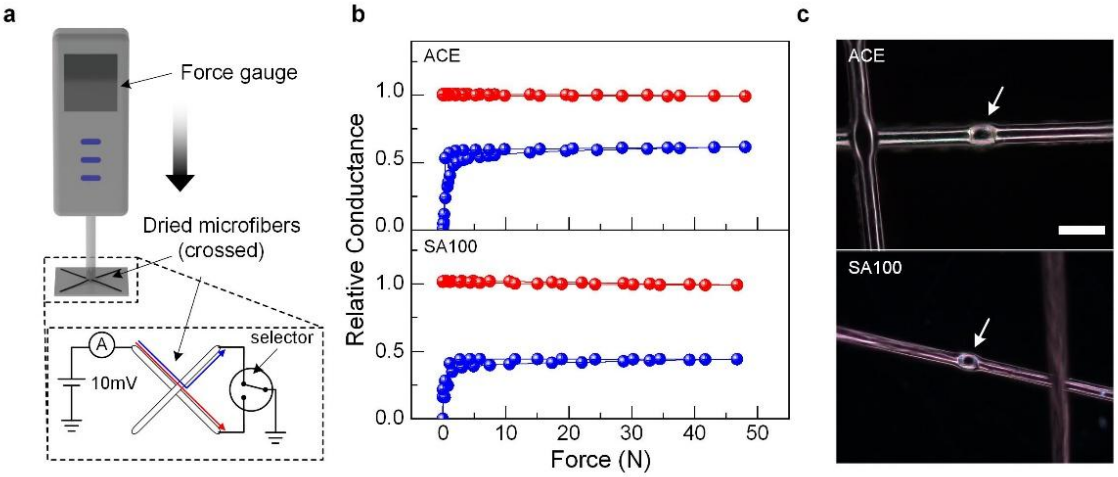

Figure S4. (a) Experimental scheme of measuring current through a single microfiber (red, direct current) and two microfibers in contact under force (blue, crossed current) in the dry state. (b) Measured currents using ACE (upper) and SA100 single fiber and two fibers. (c) Polarized optical microscopy images of PEDOT:PSS fibers after physically pressed: ACE (upper) and SA 100 (lower). Scale bar denotes 50 μm.

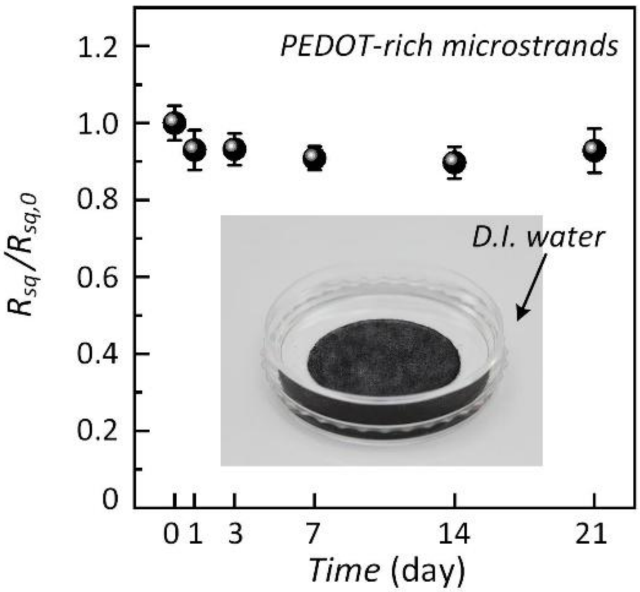

Figure S5. The plot of relative sheet resistance of mesh electrodes after dipping in water for 1, 3, 7, 14, and 21 days. Inset shows the photograph of self-fused PEDOT:PSS fiber mesh immersed in deionized water.

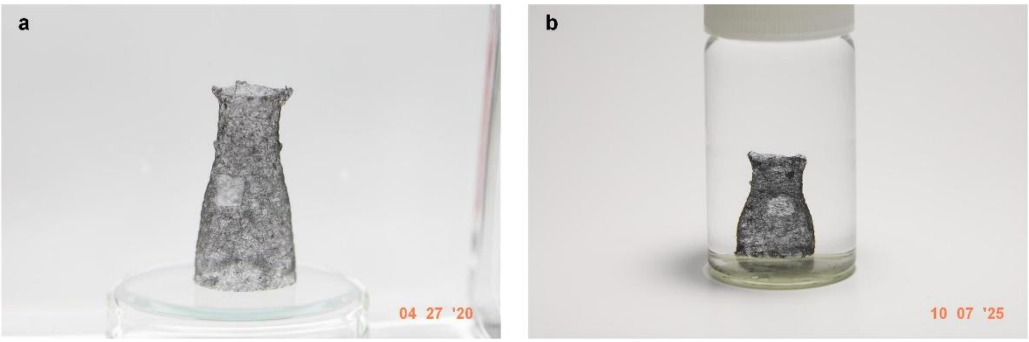

Figure S6. Photograph of the 3D PEDOT:PSS microfiber structure which was fabricated more than 5 years ago.

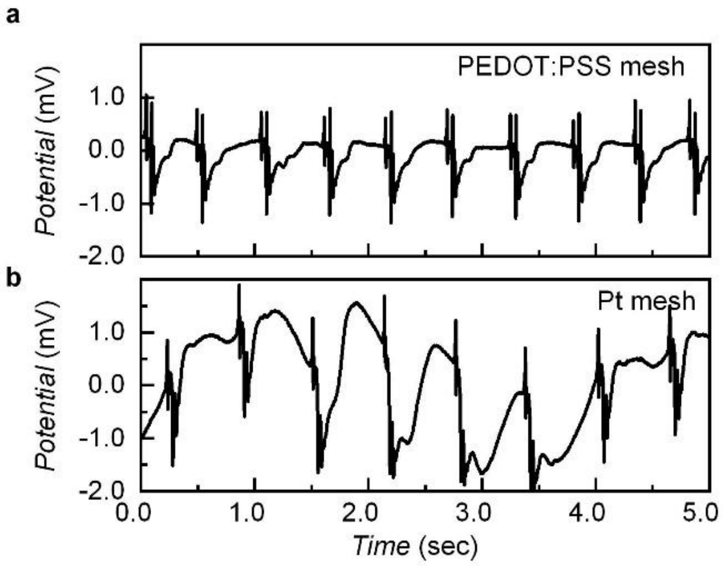

Figure S7. Electrophysiological signals recorded from (a) PEDOT:PSS electrodes and (b) conventional electrodes without Butterworth filtering.

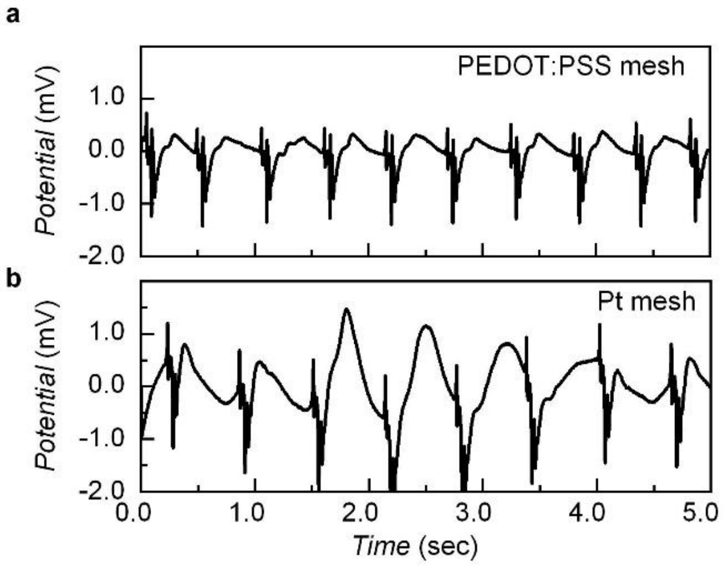

Figure S8. Electrophysiological signals recorded from (a) PEDOT:PSS electrodes and (b) conventional electrodes with Butterworth filtering.
